# Comparison of adverse perinatal outcomes between Asians and Caucasians: a population-based retrospective cohort study in Ontario

**DOI:** 10.1186/s12884-020-03467-w

**Published:** 2021-01-05

**Authors:** Na Zeng, Erica Erwin, Wendy Wen, Daniel J. Corsi, Shi Wu Wen, Yanfang Guo

**Affiliations:** 1 School of Epidemiology and Public Health, University of Ottawa, Ottawa, Ontario Canada; 2 OMNI Research Group, Clinical Epidemiology Program, Ottawa Hospital Research Institute, Ottawa, Ontario Canada; 3 Better Outcomes Registry & Network Ontario, Children’s Hospital of Eastern Ontario, Ottawa, Ontario Canada; 4 Ottawa Hospital Research Institute, Ottawa, Ontario Canada; 5 Children’s Hospital of Eastern Ontario Research Institute, Ottawa, Ontario Canada; 6 Department of Obstetrics and Gynecology, University of Ottawa Faculty of Medicine, Ottawa, Ontario Canada

## Abstract

**Background:**

Racial disparities in adverse perinatal outcomes have been studied in other countries, but little has been done for the Canadian population. In this study, we sought to examine the disparities in adverse perinatal outcomes between Asians and Caucasians in Ontario, Canada.

**Methods:**

We conducted a population-based retrospective cohort study that included all Asian and Caucasian women who attended a prenatal screening and resulted in a singleton birth in an Ontario hospital (April 1st, 2015-March 31st, 2017). Generalized estimating equation models were used to estimate the independent adjusted relative risks and adjusted risk difference of adverse perinatal outcomes for Asians compared with Caucasians.

**Results:**

Among 237,293 eligible women, 31% were Asian and 69% were Caucasian. Asians were at an increased risk of gestational diabetes mellitus, placental previa, early preterm birth (< 32 weeks), preterm birth, emergency cesarean section, 3rd and 4th degree perineal tears, low birth weight (< 2500 g, < 1500 g), small-for-gestational-age (<10^th^ percentile, <3^rd^ percentile), neonatal intensive care unit admission, and hyperbilirubinemia requiring treatment, but had lower risks of preeclampsia, macrosomia (birth weight > 4000 g), large-for-gestational-age neonates, 5-min Apgar score < 7, and arterial cord pH ≤7.1, as compared with Caucasians. No difference in risk of elective cesarean section was observed between Asians and Caucasians.

**Conclusion:**

There are significant differences in several adverse perinatal outcomes between Asians and Caucasians. These differences should be taken into consideration for clinical practices due to the large Asian population in Canada.

## Background

Racial disparities in health outcomes have been widely recognized [1]. Maternal race provides a significant axis for studies investigating perinatal outcomes, including stillbirth, preterm delivery, gestational diabetes mellitus (GDM), preeclampsia, and low or high birth weight [2, 3]. For example, White women are about one and half times more likely to have preterm birth and almost three times more likely to delivery very preterm birth compared to Black women [4].

Infant mortality rate in Blacks is also doubled as compared to Whites [3]. Racial disparities in perinatal outcomes have been believed due to the complexities of social, genetic and environmental factors [1, 5, 6]. Racial disparities in access to health care and prenatal care as well as insurance coverage are also demonstrated to contribute to differential health outcomes [7–10]. A previous study noted that women belonging to a racial/ethnic-minority were more likely to be exposed to chronic stressors in their lifetime, enhancing their risk for poor perinatal outcomes [11]. Disparities in perinatal outcomes between Black and Caucasian women have been well documented by a series of studies in the United States (US) [4, 12, 13]. However, health disparities between Asian-Caucasian Americans have been understudied although Asian Americans account for 5.7% of the US population [14], which has promoted more research in the perinatal field to Asian Americans [15]. In Canada, population-based studies examining Asian-Caucasian differences in components of maternal and neonatal outcomes are scarce. Although Canada and the US share some social and economic similarities, results of studies conducted in the US may not be generalizable to the Canadian population due to differing racial composition and population context.

Asian Canadians comprise the largest and fastest growing minority group in Canada. Ontario, as the most populous province of Canada and with Asian origin accounting for 23% of the total population [16], provides a unique opportunity to investigate variations in adverse perinatal outcomes between Asians and Caucasians. We therefore conducted a retrospective cohort study to examine disparities in adverse perinatal outcomes between Asians and Caucasians in Ontario.

## Methods

### Study design and data source

The study design is a population-based retrospective cohort study. We used data from Better Outcomes Registry & Network (BORN) Ontario birth registry, which contains comprehensive perinatal information covering virtually all hospital deliveries in Ontario, to conduct this study. Data access to the BORN is managed under the Personal Health Information Protection Act, 2004 (PHIPA) [17]. This study received ethical approval from the Children’s Hospital of Eastern Ontario Research Ethics Board (16/119X) and the Ottawa Health Science Network Research Ethics Board (20160780-01H).

### Study population

All Asian and Caucasian women who attended a prenatal screening and resulted in a singleton birth in any Ontario hospital from April 1^st^, 2015 to March 31^st^, 2017, were included in this study. If participants had multiple births during the study period, only the first birth was included. We excluded women with missing information on ethnicity or classified as mixed or other racial groups. Women with a history of hypertension were excluded for analysis of gestational hypertension and preeclampsia. Women who were diagnosed with diabetes prior to the index pregnancy were excluded for analysis of GDM.

### Outcome measures

Outcome measures considered in this study consist of a range of adverse maternal and neonatal complications. Maternal outcomes included GDM, gestational hypertension, preeclampsia, placental previa, preterm birth (< 37, < 34, < 32 weeks), spontaneous preterm birth, cesarean section (elective, emergency), assisted vaginal delivery, episiotomy, and 3rd and 4th degree perineal tears. Neonatal outcomes included sentinel congenital anomalies, low birth weight (LBW) (< 2500 g, < 1500 g), macrosomia (> 4000 g), small-for-gestational-age (SGA) neonates (defined as <10^th^ percentile of birth weight for gestational age) [13], SGA neonates (<3^rd^ percentile), large-for-gestational-age (LGA) neonates (defined as >90^th^ percentile of birth weight for gestational age), 5-min Apgar score < 7, cord arterial PH ≤7.1, hyperbilirubinemia require treatment (limiting to live births), and neonatal intensive care unit (NICU) admission. Values of birth weight outside of the range of 250 g–6000 g and values of arterial cord pH outside of the range of 6.6–7.4 were considered as outliers and were set to missing.

### Exposure and covariates

Maternal race (Asian/Caucasian) was the main exposure measure, self-reported and recorded by care providers at the prenatal screening. We considered a series of relevant factors which could be potential confounders for the association between maternal race and perinatal outcomes, including maternal age at delivery(≤18,19-24,25-29,30-34,35–39, ≥40 years) [1, 2, 18–20], neighbourhood household income (lowest, 2nd, 3th, 4th, highest), parity(0, ≥1) [1, 18, 19, 21], pre-existing physical health problems (hypertension or diabetes or heart disease or pulmonary disease) [1], pre-existing mental health problems (a composite measure of depression and anxiety), previous cesarean section (yes, no) [1], pre-pregnancy body mass index (BMI) (defined as height in kilograms (kg) divided by weight in meters squared(m^2^) (< 18.5, 18.5–24.9, 25.0–29.9, 30–34.9, 35–39.9, ≥40 kg/m^2^) [1, 7], assisted reproductive technology (ART) (yes, no) [1], substance use/alcohol exposure/smoking during pregnancy (yes, no) [1, 7, 8, 20], maternal residence area (rural, urban) [2, 18], obstetrician in antenatal care team (yes, no) [2, 7, 18, 19], and hospital level of maternal care at delivery (I, II, III) [22]. We derived neighbourhood household income and maternal residence area data from the 2011 Canadian census using Statistics Canada’s Postal Code Conversion File (PCCF) through the maternal residence postal code because BORN does not collect data on social economic status [23].

### Statistical analysis

We first compared baseline characteristics between Asians and Caucasians. Continuously distributed variables were presented by mean ± standard deviation (SD) and compared by using a t-test. Categorical variables were displayed by counts and percentages and compared by using a chi-square test. We then compared adverse perinatal outcomes between Caucasians and Asians. Generalized estimating equations (GEE) model with a log link function and a Poisson distribution were used to estimate the adjusted relative risks (aRR) and adjusted risk difference (aRD) with their 95% confidence intervals (CI) of perinatal outcomes for Asians, with Caucasians as the reference [24, 25]. Potential confounding variables included in the GEE models were maternal age at delivery, neighborhood household income, pre-existing physical health problems, pre-existing mental health problems, previous caesarean section, pre-pregnancy BMI, parity, ART, substance use/alcohol exposure/smoking during pregnancy. For procedure-related outcomes (assisted vaginal delivery, episiotomy, cesarean section, episiotomy, 3rd and 4th degree vaginal tears, NICU admission) were further adjusted for maternal residence area, obstetrician in antenatal care team, and hospital level of maternal care at delivery, in addition to the aforementioned covariates. Confounders are carefully selected to be adjusted in the multivariable models for each perinatal outcome separately to avoid the occurrence of overadjustment [26, 27]. All confounders in the multivariate regression analysis were selected by ensuring they are independently associated with both race in our source population and perinatal outcomes among Caucasian women (the reference group in this study) only with a cutoff of 0.05 [28]. Multiple imputation method was used to account for missing data in the regression analysis, in which five datasets were imputed by using fully conditional specification (FCS) logistic regression method [29–31], assuming a joint distribution for all variables. Specifically, linear regression model was used for maternal age and pre-pregnancy BMI (kg/m^2^). Generalized logit model was used for household income quintile, parity, previous caesarean section, assisted reproductive technologies, substance use during pregnancy, mental health, and urban/rural residence. All variables used in multivariable analysis were included in imputation models. Statistical Analysis System (SAS) for windows, version 9.4 (SAS Institute, Cary, NC) was used to perform all of the analysis in this study, the criteria for statistical significance was set at alpha = 0.05.

## Results

**Fig. 1:**
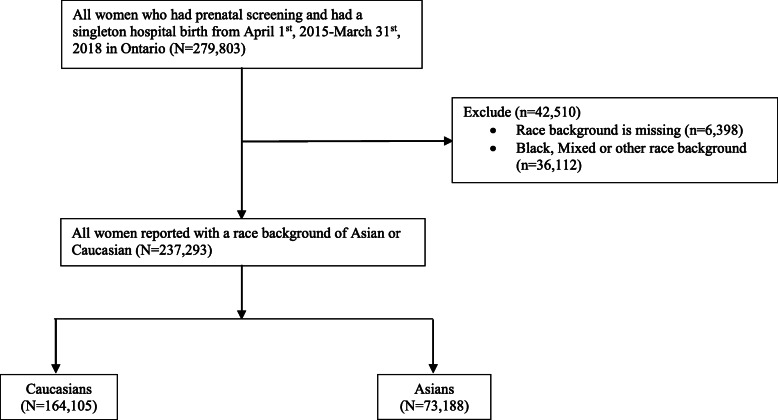
Flow chart of study population and analysis population

A total of 237,293 eligible women (30.9% Asians and 69.1% Caucasians) were included in the final analysis (Fig. 1). Compared to Caucasian women, Asian women tended to be older and had a significantly higher rate of being in the lowest income quintile level, living in urban areas, being underweight, having a previous caesarean section, and having an obstetrician on the antenatal care team. On the other hand, Asian women were less likely to be nulliparous, be overweight/obese, partake in alcohol consumption/substance use/smoking during pregnancy, have pre-existing disease, have mental health problems, and also less likely to deliver in alower maternal level of care hospital (Table 1).

**Table 1:** Comparison of characteristics between Asians and Caucasians, Ontario, Canada, April 1st, 2015-March 31st, 2017 (*N* = 237,293)

Characteristics	Asian		Caucasian		***P*** value
	n	%	n	%	
	73,188	30.9	164,105	69.1	
Maternal Age at delivery (years) (Mean ± SD)	32.07 ± 4.5		31.08 ± 4.98		<.0001
≤ 18	49	0.1	1221	**0.7**	< 0.001
19–24	3207	4.4	15,229	**9.3**	
25–29	17,792	24.3	41,652	**25.4**	
30–34	30,428	**41.6**	65,448	39.9	
35–39	18,019	**24.7**	34,207	20.9	
≥ 40	3592	**4.9**	6116	3.7	
Missing	101	0.1	232	0.1	
Neighbourhood median household income quntiles (link to 2011 Canadian Census data)					<.0001
Quintile 1 (lowest)	17,117	**23.6**	26,877	16.6	
Quintile 2	16,540	**22.8**	30,678	18.9	
Quintile 3	16,544	**22.8**	33,781	20.8	
Quintile 4	13,679	18.9	37,563	**23.2**	
Quintile 5 (highest)	8556	11.8	33,148	**20.5**	
Missing	752	1.0	2058	**1.3**	
Maternal pre-existing disease^a^					<.0001
No	70,579	**96.4**	151,491	92.4	
Yes	2609	3.6	12,544	**7.6**	
Mental health Condition					<.0001
No	66,363	96.6	123,975	80.8	
Yes	2366	3.4	29,465	**19.2**	
Missing	4459	6.1	10,665	6.5	
Previous cesarean section					
Yes	60,129	83.8	138,671	**86.5**	<.0001
No	11,656	16.2	21,624	13.5	
Missing	1403	1.9	3810	2.3	
Pre-pregnancy BMI (kg/m^2^) (Mean ± SD)	23.4 ± 4.5		25.74 ± 6.17		<.0001
Underweight (< 18.5)	5958	**9.7**	6537	4.4	<.0001
Normal (18.5–24.9)	36,655	**59.7**	75,072	50.8	
Overweight (25.0–29.9)	13,409	21.8	36,235	**24.5**	
Obese (30–34.9)	3982	6.5	17,044	**11.5**	
Obese (35–39.9)	1001	1.6	7982	**5.4**	
Obese (≥40)	378	0.6	4991	**3.4**	
Missing	11,805	16.1	16,244	9.9	
Parity					<.0001
0	31,289	43.1	75,996	**46.7**	
≥ 1	41,386	**56.9**	86,738	53.3	
Missing	513	0.7	1371	0.8	
Conception by assisted reproductive technology					<.0001
No	65,565	96.2	145,616	95.4	
Yes	2622	3.8	6978	**4.6**	
Missing	5001	6.8	11,511	7.0	
Drug use during pregnancy					<.0001
No	69,375	97.9	137,147	86.7	
Yes	1458	2.1	21,012	**13.3**	
Missing	2355	3.2	5946	3.6	
Alcohol exposure during pregnancy					<.0001
No	69,833	99.1	152,805	97.2	
Yes	612	0.9	4470	**2.8**	
Missing	2743	3.7	6830	4.2	
Smoking during pregnancy (any time)					<.0001
No	69,485	98.9	140,296	89.4	
Yes	771	1.1	16,575	**10.6**	
Missing	2932	4.0	7234	4.4	
Maternal residence area					
Urban	72,386	**99.2**	139,566	85.4	
Rural	593	0.8	23,841	**14.6**	
Missing	209	0.3	698	0.4	
Obstetrician in antenatal care team					<.0001
No	11,171	15.3	49,579	30.2	
Yes	62,017	**84.7**	114,456	69.8	
Hospital level of maternal care					< 0.001
level I	1383	1.9	18,597	**11.3**	
level II	60,006	**82.0**	101,777	62.0	
level III	11,799	16.1	43,661	**26.6**	

^a^Maternal pre-existing disease includes any of hypertension, diabetes, heart disease, and pulmonary disease

1. Missing data represents missing values for neighborhood household income level and education level, parity, previous caesarean section, drug use, alcohol use, birth weight and antenatal health care provider were excluded from the percentage calculation

2. Bold values mean the risk factor favouring corresponding race group

Compared with Caucasian women, Asian women had higher risks of GDM, placental previa, preterm birth (< 37, < 34, < 32 weeks), spontaneous preterm birth, emergency cesarean section, episiotomy and 3rd and 4th degree perineal tears, but lower risks of gestational hypertension, preeclampsia after adjusting for relevant confounders (Table 2). No difference was found in risk of elective caesarean section between these two groups.

**Table 2:** Comparison of risks of adverse maternal outcomes between Asians and Caucasians, Ontario, Canada, April 1^st^, 2015-March 31^st^, 2017 (*N* = 69,734)

Maternal Outcomes	Asian		Caucasian (reference)		Adjusted RR (95% CI)	Adjusted RD (95%CI)
	n	%	n	%		
Gestational diabetes	9793	13.38	9077	5.53	2.71 (2.68, 2.74)	1.00 (0.97, 1.03)
Gestational hypertension	2012	2.75	6461	3.94	0.93 (0.88, 0.98)	−0.07 (− 0.12, − 0.02)
Preeclampsia	1945	2.66	7045	4.29	0.84 (0.78, 0.89)	−0.18 (− 0.23, − 0.13)
Placental previa	700	0.96	1097	0.67	1.30 (1.21, 1.40)	0.26 (0.17, 0.36)
Preterm birth (< 37 weeks)	5070	6.93	10,419	6.35	1.23 (1.20, 1.27)	0.21 (0.17, 0.24)
Preterm birth (< 34 weeks)	1376	1.88	2603	1.59	1.37 (1.29, 1.44)	0.31 (0.24, 0.38)
Preterm birth (< 32 weeks)	901	1.23	1582	0.96	1.49 (1.39, 1.58)	0.40 (0.30, 0.49)
Spontaneous preterm birth	1994	2.72	4103	2.50	1.25 (1.20, 1.31)	0.23 (0.17, 0.28)
Cesarean section	21,694	29.64	46,416	28.30	1.03 (1.01, 1.05)	0.03 (0.01, 0.05)
Elective cesarean section	11,542	15.77	24,968	15.22	0.91 (0.89, 0.94)	−0.09 (−0.11, −0.07)
Emergency cesarean section	10,146	13.86	21,443	13.07	1.22 (1.20, 1.25)	0.20 (0.18, 0.22)
Assisted vaginal delivery	8521	11.64	14,601	8.90	1.29 (1.26, 1.31)	0.25 (0.23, 0.28)
Episiotomy	10,443	14.27	14,552	8.87	1.40 (1.38, 1.43)	0.34 (0.31, 0.36)
3^rd^ and 4^th^ degree perineal tears	2756	4.19	3836	2.65	1.57 (1.52, 1.62)	0.45 (0.40, 0.50)

*RR* relative risk, *CI* confidence interval

1. Generalized estimating equations with a log link function and a poisson distribution were used to estimate the relative risks of the outcomes.

2. Covariates included in the adjusted models for each outcome were selected covariates that showed univariate association of *P* < 0.05 with both the exposure and the outcome were included in the adjusted model. The covariate for each outcome was fit separately

3. A fully conditional specification method was used to impute missing values, assuming a joint distribution for all variables. Five imputed datasets were created

Compared with Caucasians, Asians had higher risks of low birth weight (< 2500 g, < 1500 g), SGA neonates (<10th percentile, <3rd percentile), NICU admission, and hyperbilirubinemia requiring treatment, but had lower risks of sentinel congenital anomalies, macrosomia, LGA neonates, 5-min Apga score < 7, and arterial cord pH ≤7.1 after adjusting potential confounders (Table 3).

**Table 3:** Comparison of risks of adverse neonatal outcomes between Asians and Caucasians, Ontario, Canada, April 1^st^, 2015-March 31^st^, 2017 (*N* = 69,734)

Neonatal outcome	Asian		Caucasian (reference)		Adjusted RR (95% CI)	Adjusted RD (95%CI)
	n	%	n	%		
Sentinel Congenital Anomalies	1046	1.43	2938	1.79	0.90 (0.83, 0.98)	−0.10 (−0.18, −0.03)
Low birth weight (< 2500 g)	5089	7.00	7160	4.40	1.81 (1.77, 1.85)	0.59 (0.55, 0.63)
Low birth weight (< 1500 g)	756	1.04	1229	0.76	1.59 (1.49, 1.69)	0.46 (0.36, 0.56)
Macrosomia (> 4000 g)	3459	4.76	19,214	11.80	0.43 (0.39, 0.46)	−0.85 (−0.89, − 0.82)
Small-for-gestational-age neonates (<10^th^ percentile)	10,396	14.35	12,229	7.56	1.93 (1.91, 1.96)	0.66 (0.63, 0.69)
Small-for-gestational-age neonates (<3^rd^ percentile)	2703	3.73	2879	1.78	2.19 (2.14, 2.25)	0.78 (0.73, 0.84)
Large-for-gestational-age neonates (>90^th^ percentile)	3610	4.98	17,660	10.92	0.50 (0.46, 0.53)	−0.70 (−0.73, −0.66)
5- min Apgar score < 7	1219	1.69	3742	2.32	0.89 (0.82, 0.96)	−0.11 (− 0.18, − 0.05)
Arterial cord pH ≤ 7.1	2595	4.04	8704	6.11	0.71 (0.66, 0.76)	−0.34 (− 0.39, − 0.30)
NICU admission	8529	11.65	18,796	11.46	1.18 (1.16, 1.21)	0.17 (0.14, 0.19)
Hyperbilirubinemia requiring treatment	4300	6.95	8132	5.52	1.41 (1.37, 1.45)	0.35 (0.31, 0.38)

*RR* relative risk, *CI* confidence interval, *RD* risk difference, *NICU* neonatal intensive care unit

1. Generalized estimating equations with a log link function and a poisson distribution were used to estimate the relative risks of the outcomes.

2. Covariates included in the adjusted models for each outcome were selected covariates that showed univariate association of *P* < 0.05 with both the exposure and the outcome were included in the adjusted model. The covariate for each outcome was fit separately

3. A fully conditional specification method was used to impute missing values, assuming a joint distribution for all variables. Five imputed datasets were created

## Discussion

In this population-based study, we have several principal findings. First, we found that compared with Caucasians, Asians had elevated risks of GDM, placental previa, preterm birth, and emergency cesarean section, whereas had lower risks of gestational hypertension, preeclampsia. Second, Asians have elevated risk of LBW, SGA, NICU admission, and hyperbilirubinemia requiring treatment, compared to Caucasians, but are less likely to have macrosomia, LGA, 5-min Apgar score < 7, sentinel congenital anomalies, and arterial cord pH ≤ 7.1. We find no difference in risk of elective cesarean section was observed between the two groups.

The most substantial difference in adverse maternal outcomes between Asian and Caucasian women observed in this study was GDM. Asian background has been associated with markedly increased risk of GDM [32], and our study finding provided additional evidence supporting an increased risk of GDM in Asians. A recent systematic review and meta-analysis showed that the pooled rate of GDM in Asians was 11.5% (95% CI 10.9–12.1) [33], which is close to the rate of GDM (13.7%) in Asians in this study. Another important difference in adverse maternal outcomes observed in this study was that Asian women had a higher rate of 3rd and 4th degree perineal tear than Caucasian women, which is consistent with previous studies [34, 35]. In our study, Asian women were less likely to have macrosomic babies and less likely to be nulliparous, which are protective against perineal tears [36]. This phenomenon is likely associated with the smaller stature and shorter perineum of Asian women relative to Caucasian women [37]. Asian women had higher risk of preterm birth, which is similar to previous studies [38, 39]. This is more pronounced in the subgroup of early preterm birth (e.g.,< 32 weeks). The findings of Asians having an elevated risk of placenta previa is also consistent with our previous studies [40] which may be explained by cultural influence (such as stress), nutrition or true genetic differences. Asian women are observed to have a relatively larger placenta even though the reasons are still not elucidated [41]. The higher rate of emergency cesarean section among Asians may be explained by social deprivation or communication difficulties [42]. We speculate that the lower risk of preeclampsia and gestational hypertension for Asian women, may in part be explained by their lower risk of health behaviours such as recreational drugs, alcohol, and cigarette smoking during pregnancy that was observed in this study and many previous studies [43–45], although it is still unclear.

The most striking difference in neonatal outcomes between Asians and Caucasians was the size of the newborns, in which Asians had higher risks of low birth weight and SGA but lower risk of macrosomia and LGA than Caucasians. These findings are in general consistent with previous studies [20, 21, 46, 47], although somewhat different from an earlier study by our team comparing birth weight distribution between Caucasian and East Asian (Chinese) [48]. Specifically, we found that while the mean birth weight in Chinese was substantially lower than that of in Caucasians with lower rate of macrosomia, the rate of low birth weight was also lower in Chinese infants [48]. As we have stated earlier, Asians in this study were from different regions in Asia with some distinctive features and lumping them together limited our ability to identify specific differences among them.

The higher rate of NICU admission for neonates delivered by Asian women might be associated with the increased risk of early preterm birth among Asian women found in our study. The result of lower rate of 5-min Apgar score < 7 among Asian population compared with Caucasian in our study, which is similar to a CDC US report covering 3,163,441 live births from 48 Reporting States and the District of Columbia [49]. It is also consistent with results from a prior study (monthly vital statistics report_1995) [50]. However, the reasons for lower rate of 5-min Apgar score < 7 are still needed to be explored.

The finding of higher risk of hyperbilirubinemia required treatment in Asian infants was consistent with previous studies [51, 52]. One of the reasons the phenomenon of increased risk of hyperbilirubinemia required treatment among Asians may be caused by a common DNA-sequence variant (Gly71Arg) carried by Asians, resulting in an amino acid change in the uridine diphosphate glucuronosyl-transferase protein [53]. Asian women were found to have a slightly lower risk of sentinel congenital anomalies, whereas a previous study found no difference in overall congenital anomalies (including sentinel congenital anomalies, down syndrome etc.) between Asians and Caucasians [39].

There are several strengths of this study. First, this study is based on a large population with comprehensive demographic and health care information, allowing an investigation of a number of adverse perinatal outcomes with appropriate adjustment for potential confounding factors. Second, our study has a large sample size of Asian women, enabling a robust comparison between Caucasians and Asians with greater than at least 90% power to detect the difference for each perinatal outcome with a two-tailed alpha (type 1 error) of 5%, where previous studies had smaller samples of Asians [1, 7, 18, 54, 55]. Third, universal access to quality maternity care helped to isolate maternal factors from health care factors.

Limitations of this study should be acknowledged. First, Asians in this study included women from a variety of regions in Asia. Although these women share some common demographic and cultural background, there are major differences in genetic and environmental factors among them. However, although grouping different Asians together may have limited our ability to reveal some specific differences from Caucasians and to properly interpret specific results, it gives us an overall sense of discrepancies in perinatal outcomes between Asians and Caucasians, which will direct us to focus on some specific outcomes in future work. Second, as race statue is considered to be a subjective assessment, which might generate misclassification of race status leading to unavoidable bias. Third, as our study population included women who had undergone prenatal screening so that it only captures approximately 70% of pregnant women in Ontario [56]. Women who attend prenatal screening tend to live in an urban area and high income neighbourhood, to receive prenatal care from an obstetrician, and are more likely be an immigrant or a refugee [57]. Fourth, there were significant differences in baseline characteristics between Asians and Caucasians, which might still have impact on our results due to possible residual confounding. Finally, we did not cover some perinatal outcomes, including placenta accreta, postpartum haemorrage, neonatal asphyxia and infection in our study due to incomplete information in BORN database, and did not report some underpowered outcomes, such as maternal ICU admission, placental abruption, stillbirth, 5-min Apgar score < 4 and neonatal death due to their low incidence rates (less than 1% in Ontario). Despite these limitations, the results of this study are valuable in informing future work on perinatal outcomes in persons from subgroups within the Asian diaspora.

## Conclusion

In summary, our population-based study found significant differences in several adverse perinatal outcomes between Asians and Caucasians. Given the heterogeneity in the demographic and social characteristics among different Asian groups, future studies will be valuable to explore these differences among specific Asian groups.

## Data Availability

The data analyzed during this study are held securely at the prescribed registry BORN Ontario. Data sharing regulations prevent these data from being made available publicly due to the personal health information in the datasets. Enquiries regarding BORN data must be directed to BORN Ontario (Science@BORNOntario.ca).

## Abbreviations

ART: Assisted Reproductive Technology
BMI: Body Mass Index
BORN: Better Outcomes Registry & Network
CI: Confidence Interval
FCS: Fully Conditional Specification
GDM: Gestational Diabetes Mellitus
GEE: Generalized Estimating Equations
LGA: Large-for-Gestational-Age
NICU: Neonatal Intensive Care Unit
PCCF: Postal Code Conversion File
PHIPA: Personal Health Information Protection Act
RD: Risk Difference
RR: Relative Risk
SAS: Statistical Analysis System
SD: Standard Deviation
SGA: Small-for-Gestational-Age
US: United States
